# 12- and 24-Month-Old Infants’ Search Behavior Under Informational Uncertainty

**DOI:** 10.3389/fpsyg.2020.00566

**Published:** 2020-03-27

**Authors:** Sunae Kim, Beate Sodian, Joëlle Proust

**Affiliations:** ^1^Department of Developmental and Clinical Child Psychology, Institute of Psychology, Eötvös Loránd University, Budapest, Hungary; ^2^Developmental and Educational Psychology, Ludwig-Maximilian University of Munich, Munich, Germany; ^3^Institut Jean-Nicod, Department of Cognitive Science, Ecole Normale Supérieure, Paris, France

**Keywords:** infant uncertainty, searching, probabilistic information, subjective uncertainty, latency

## Abstract

Infants register and react to informational uncertainty in the environment. They also form expectations about the probability of future events as well as update the expectation according to changes in the environment. A novel line of research has started to investigate infants’ and toddlers’ behavior under uncertainty. By combining these research areas, the present research investigated 12- and 24-month-old infants’ searching behaviors under varying degree of informational uncertainty. An object was hidden in one of three possible locations and probabilistic information about the hiding location was manipulated across trials. Infants’ time delay in search initiation for a hidden object linearly increased across the level of informational uncertainty. Infants’ successful searching also varied according to probabilistic information. The findings suggest that infants modulate their behaviors based on probabilistic information. We discuss the possibility that infants’ behavioral reaction to the environmental uncertainty constitutes the basis for the development of subjective uncertainty.

## Introduction

The environment is full of uncertainty. Importantly, not all events are completely unpredictable. Some information and events have a higher likelihood of occurrence than others. An ability to accurately predict a future event and optimally behave accordingly is crucial for survival. Here, we investigated infants’ searching behavior for a hidden object under circumstances where infants were faced with given probabilistic information about a hiding location. Specifically, we examined how informational uncertainty influences infants’ search latency (time to initiate searching).

Infants around 12 months register and react to uncertainty. A prime example is well demonstrated by “social referencing” (e.g., Campos and Stenberg, 1981; Sorce et al., 1985; Boccia and Campos, 1989; Walle et al., 2017; for a review). In these studies, infants facing an uncertain situation regulate their own behavior by resorting to others’ emotional reaction. Using a classical visual cliff study, when faced with a drop-off on thePlexiglas surface, infants regulate their behavior (either to crawl over the apparent drop-off in the middle of the Plexiglas or stay away from it) depending on whether their caregivers in the opposite side of the surface express positive or negative/fearful emotions (Sorce et al., 1985). Infants display social referencing behaviors as an epistemic appraisal of the situation rather than as a mere low-level reaction (Stenberg, 2003, 2009).

Moreover, infants form predictions informed by prior learning or knowledge (Paulus et al., 2011; Emberson et al., 2015) and to further adjust their predictions according to the changes in the external information inputs (Kayhan et al., 2019). Finally, empirical studies demonstrate that young infants have some appreciation of the probabilities of events in their environment (e.g., Téglás et al., 2007, 2011; Xu and Garcia, 2008; Denison et al., 2013). In Téglás et al. (2007), 12-month-old infants were presented with a movie where three identical objects and one object differing in color and shape randomly bounced in a round container that had a bottom opening. Infants looked longer at an improbable event (in which the different object exited at the bottom opening) than a probable event (in which one of the three identical objects exited at the bottom opening). This behavior was not due to infants’ mere reaction to a salient feature of the event (Experiment 2). Thus, infants as young as 12 months old form expectations about a probabilistic future event without observing actual event frequencies.

While these studies demonstrate infants’ ability to learn and appreciate the informational input of their environment, they do not directly address the question of whether and how infants use probabilistic information to guide their future behavior. Social referencing suggests that infants around 12 months old are already able to adopt either an avoiding or approaching behavior by relying upon others’ (caregivers) reaction toward uncertain situations. However, it remains to be known whether they react differently according to the uncertainty level without an adult’s help.

A few recent studies provide some insight into infants’ and young toddlers’ behaviors under informational uncertainty. In one study, Call and Carpenter (2001) tested 30-month-old infants’ searching behaviors (Experiment 3). A sticker was hidden in one of three tubes; in some trials the hiding was done in front of the infants while in other trials the infants’ view to the hiding was blocked. Infants looked into the tubes (i.e., bending their heads) more frequently (before choosing the tube) when they had not seen the hiding than when they had seen it. Goupil and her colleagues demonstrated that infants are more likely to seek for help (Goupil and Kouider, 2016) and less likely to persistently search for a hidden object (Goupil et al., 2016) when information is uncertain than when it is certain. In Goupil et al. (2016), 20-month-old infants saw an object being placed inside one of the two containers. Infants were asked to point to the box that contained the hidden object after varying amount of delay and they were given the box of their choice to retrieve the object. With an increasing delay, children were less accurate in pointing at the location of the hidden object. Critically, another group of infants were trained to use help from their parent to be given access to the relevant box. This group, compared to the no-training group, was more likely to accurately retrieve the hidden toy. Therefore, infants react differently under uncertain situations by gathering more information – either asking for help (Goupil et al., 2016) or exploring by themselves (Call and Carpenter, 2001; Goupil and Kouider, 2016). These studies nicely demonstrate how infants appreciate and deal with an uncertain environment and have been argued to suggest early metacognitive sensitivity.

In the present research, we attempted to combine these lines of research and asked whether and how young infants differently react under a varying degree of informational uncertainty. Drawing on the literature on visual search under perceptual variability and uncertainty (see Eckstein, 2011, for a review, see also Weidemann and Kahana, 2016), we used a temporal measurement to assess how certainty of information might influence infants’ production of searching. Infants’ time to initiate searching was measured by delay of infants’ touching any container. We manipulated across task types the probabilistic information of the location of a hidden toy. We expected that, with increasing informational uncertainty of the object location, infants would take an increasingly longer time to initiate searching. We also assessed infants’ successful searching across uncertainty levels. Finally, we assessed the relationship between search onset delay and performance. We tested 24-month-olds in order to establish a phenomenon of our research question as the design was novel. We also tested 12-month-olds in order to investigate whether infants at such a young age show a similar pattern as 24-month-olds.

## Materials and Methods

### Participants

Twenty-four-month old (*N* = 39, 19 females, 21 males, mean age = 2.03, range = 1.84 ∼ 2.24) and 12-month-old (*N* = 30, 13 females, 17 males, mean age = 1.03, range = 0.94 ∼ 1.08) participated in the experiment. Additional 28 infants were tested but excluded for the final analyses due to major experimental errors (e.g., repeating the same trial, missing a trial, or forgetting to add containers *n* = 22), infants’ fussiness (*n* = 5), or an infant’s lack of his motor skill (*n* = 1). This number also included six infants during a pilot phase in which different sizes of a screen and containers were used before concluding the stimuli. All infants were white European descendants except for one 24-month-old Asian-European infant.

### Materials

**FIGURE 1:**
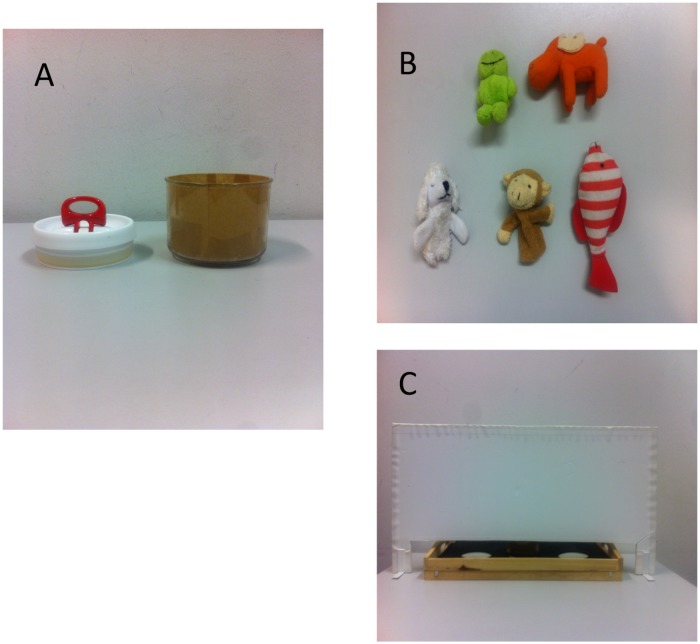
Stimuli and an experimental setting. **(A)** A lid and a container. **(B)** Toys. **(C)** A screen and a tray.

Materials included a table (804 mm × 709 mm), a tray (519 mm × 297 mm), a screen (692 mm × 395 mm), three identical containers each with a lid on top which can be easily lifted, and five different toys. Figure 1 portrays stimuli.

### Design and Procedure

All infants were tested in a laboratory at a (university masked for a blind review). They were seated at a table on a parent’s lap across an experimenter. The parent was blindfolded. Every child received two familiarization trials followed by three test trials. The first familiarization trial was designed to familiarize infants with opening a lid of a container (grabbing and lifting a lid). The experimenter demonstrated how to open the lid to retrieve a toy inside and let infants lift the lid by themselves. Opening the lid was easy to perform on the part of the tested infants. The second familiarization trial was designed to familiarize infants with a test setting involving a screen and a tray. The experimenter placed a container in the middle position of the tray and placed the screen in front of the tray, blocking only the upper half of the container. S/he then introduced a toy, holding it above the screen while lifting the lid of the container (“Hi [a child’s name]. Look what I have! It is a bear. I’m going to put this into this box.”), and placed the toy inside the container and closed the lid (“Yup! I just put it in the box. The bear is in the box.”). Finally, she removed the screen, pushed the tray toward infants, and asked infants to find the hidden toy (“Where is the bear?”). The experimental question was asked up to three times if infants did not react, each questioning being made 10 s apart. By the end of the second familiarization trial, all infants had no problem to immediately reach out and lift up the lid. The tray was always first positioned at the edge of a table where the experimenter sat and was pushed all the way toward the other side of the table where the infant sat. A few infants had a tendency to crawl over the table but we asked parents to hold them back if this happened.

Immediately after the second familiarization trial, infants received one test trial per task type. In a *one-box* task, the experimenter placed a container in one of the three positions on the tray. S/he placed the screen (blocking only the upper half of the container) in front of the tray, introduced a toy, as in the second FM trial (“Look what I have! It is a fish. I’m going to put this into this box [one of these boxes (in two-box and three-box trials)]”). She placed the toy inside the container and closed the lid as in the second FM trial (“Yup! I just put it in the box. Now the fish is in the box”). She placed two additional containers in the other two remaining positions on the tray, removed the screen, and pushed the tray toward the infants. In a *two-box* task, the experimenter initially placed two containers on the tray, placed the screen (blocking only the upper half of the container), and hid a toy into one of the containers. She placed an additional container in the remaining spot, removed the screen, and pushed the tray toward the infants. In a *three-box* task, the experimenter placed three containers on the tray, placed the screen (blocking only the upper half of the container), and hid a toy into one of the three containers. She then removed the screen and pushed the tray toward the infants. At the end of every test trial, infants were asked to find a hidden toy (“Where is the fish?”) from the three containers on the tray. In each trial a different toy was used. Order of test trials was counterbalanced across participants. The hiding position of the toy in each trial was counterbalanced across and within participants.

### Data Coding

#### Search Onset Delay

Onset delay was calculated as the duration between the time point when an experimenter pushed the tray until the infants’ hand first contacted *any* container. Two independent coders separately coded all the data. A second coder was blinded to our test hypotheses and did not know in which task type infants were tested. There was 98.5 percent reliability: all trials that were differently coded by the two coders differed in 0.1 s, in which cases an average was taken as a final data point.

#### Successful Searching

We coded whether infants found the toy in their first attempt in each trial.

We also separately coded the experimenter’s videos (those that filmed infants’ views toward the experimenter) to ensure that infants could not identify the location of the hidden toy by other cues such as a container being moved slightly while a toy being placed. We did this by having another adult guess the location of the hidden toy in two-box and three-box trials. There were six such cases but in none of these did infants accurately identify the toy’s location in their first attempt. Thus, we included them in our final data analyses.

## Results

We analyzed search onset delay via a 2 (Age: 12-month-olds vs. 24-month-olds) × 3 (task type: 1-box vs. 2-box vs. 3-box) ANOVA with Age as a between subject factor. Task type was not significant, *F*(2, 134) = 2.78, *p* = 0.066 nor was the interaction between Age and Task type, *F*(2, 134) = 0.201, *p* = 0.819. Age was significant *F*(1, 67) = 4.654, *p* = 0.035, η*^2^* = 0.065 indicating that older infants took less time to initiate searching than younger infants.

**FIGURE 2:**
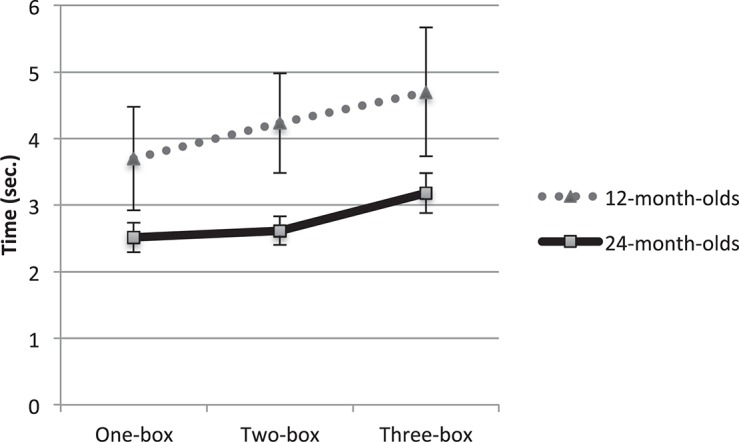
Infants’ search onset delay. A linear increase of time to initiate searching was significant for both age groups. Error bars indicate standard errors.

Importantly, we asked whether infants’ search onset delay increased across task type by conducting a planned linear contrast test. A linear increase of search onset delay across task type was significant, *F*(1, 67) = 13.448, *p* < 0.001, η*^2^* = 0.167. That is, infants took increasingly longer time to initiate searching as uncertainty increased and Age did not interact with a linear trend of Task type, *F*(1, 67) = 0.538, *p* = 0.466. Infants took longer to initiate searching in the 3-box than in the 1-box trial, *t*(68) = 3.615, *p* = 0.001; 2-box vs. 1-box: *t*(68) = 0.766, *p* = 0.446; 3-box vs. 2-box: *t*(68) = 1.24, *p* = 0.219. See Figure 2.

Next, we examined children’s successful searching in their first attempt (i.e., finding the toy in their first attempt) against chance^1^ (=0.33). Thirty-seven out of 69 (*p* < 0.001), 38 out of 69 (*p* < 0.001) and 20 out of 69 infants (*n.s.*) successfully found the toy in their first attempt in the one-box, the two-box, and the three box, respectively (binomial tests). McNemar tests were conducted to compare infants’ successful searching across task types: infants’ performance in the two box and the three box was significantly different from one another (*p* = 0.008) and one box and three box (*p* = 0.005) but did not differ between one-box and two-box trials (*p* = 1.00).

Finally, we examined the relation between search onset delay and successful searching. Thirteen infants (younger: *n* = 4; older; *n* = 9) were excluded for this analysis because they were either successful (*n* = 2) or unsuccessful (*n* = 11) in all trials. The mean onset delay was analyzed by 2 (performance: successful vs. non-successful trials) × 2 (age: 24-month-olds vs. 12-month-olds) ANOVA with Age as a between-subject factor. Only Performance was significant, *F*(1, 54) = 4.661 *p* = 0.035, η^2^ = 0.079. Infants took significantly longer time to initiate their search in unsuccessful search trials (*M* = 3.50, SD = 1.82) as compared to successful search trials (*M* = 3.00, SD = 2.07). Age was not significant, *F*(1, 54) = 1.908, *p* = 0.173 nor was an interaction of Age × Performance *F*(1, 54) = 1.267, *p* = 0.265.

Because infants received only one trial per task type we combined trials across the task type in the previous analysis. However, presumably the three task types differ in terms of their memory demand and the cognitive process involved, and thus we examined the infants’ onset delay and performance in each task type in the subsequent analyses. First, we identified the infants’ onset delay in each trial as either short or long depending on whether it is below or above their average onset delay across three trials. Those infants whose onset delays were the same in all three trials were excluded (*n* = 12). Those trials in which infants’ onset delay was equal to the average were not considered. In the one-box trial, among the infants who belonged to the short onset delay group 64% of the infants (25 out of 39) displayed successful searching whereas those infants who belonged to the long onset delay group 44% of the infants (7 out of 16) displayed successful searching, χ^2^ = 1.93, *p* = 0.16. In the two-box trial, among the short onset delay group, 68% of the infants (19 out of 28) displayed successful searching whereas among the long onset delay group, 41% of the infants (9 out of 22) displayed successful searching. χ^2^ = 3.63, *p* = 0.056. Finally, in the three-box trial, 33% of the infants (7 out of 21) displayed successful searching in the short onset group, and 21% of the infants (7 out of 34) in the long onset group did so, χ^2^ = 1.11, *p* = 0.29.

## Discussion

In the present research, infants displayed a linear increase of time delay in search onset with an increasing degree of informational uncertainty. Infants’ successful searching also varied as a function of their informational uncertainty. Finally, infants’ search onset delay was reliably differentiated between when they successfully vs. unsuccessfully searched an object. Overall, our study demonstrates that infants modulate their search behavior in light of probabilistic information.

The present findings concerning the time delay in search onset suggest that infants as young as 12 months old are sensitive to different levels of uncertainty. Given that this time measure was administered prior to the start of actual searching, search onset delay did not depend on their experience of actual event outcomes. The time infants used to initiate their searching behavior could be interpreted as reluctance or appetence. In that sense, infants’ varying degree of appetence may indicate their expectation of a toy being probably hidden in a given box – an expectation formed before engaging in searching, and reluctance inversely indicating a lack of such an expectation. Note that infants were required to keep track of an event that unfolds in time in order to predict object location, and hence, produce appropriate searching behaviors. Additionally, the three-box task was less complex than either the one-box or the two-box tasks in this respect (e.g., no additional containers being added); yet infants were slower to begin to search. Finally, in all trials infants were in the end invited to search from three identical boxes. Thus, these findings cannot be merely attributed to infants’ reaction to surface features or superficial task demands. Notably, Age did not interact with the task type suggesting that a similar pattern of responses exists in both age groups – although the 12-month-olds as compared to the 24-month-olds were slower to initiate their searching and larger variability was observed in the younger group. A similar pattern of the onset delay for the two age groups may indicate that a similar cognitive process is involved developmentally. However, a longer response latency in the younger group may indicate that the decision making process is slower in the younger than in the older group. A larger variability in the younger group may indicate that large individual differences are present in the younger group. Prior studies on infants’ appreciation of probabilistic events demonstrate that very young infants are able not only to detect and extract probabilistic information but also to predict a probabilistic future event without being exposed to event frequencies (e.g., Téglás et al., 2007). The current findings further extend prior work by demonstrating that infants as young as 12 months old *behave* according to the probabilistic information. They also extend the social referencing literature by demonstrating that infants react to different levels of uncertainty in the environment – without relying upon others.

How do our findings relate to previous work on infants’ dealing with uncertain environments? Prior work has found that infants react differently under uncertain situations by gathering more information (e.g., Call and Carpenter, 2001; Goupil et al., 2016). These findings have been interpreted as evidence that infants and young toddlers have an access to their own uncertainty and that this ability relies upon the same brain regions for error monitoring as in adults (Goupil and Kouider, 2016). Note that infants in our study did not impulsively or randomly search for a hidden toy across task types. Rather, they were increasingly slower to initiate searching as informational uncertainty increased. Especially given that searching did not entail any immediate risks – thus in the absence of a reason to avoid reaching for and obtaining a toy, a rather adaptive behavior might have prompted immediate initiations of searching. That infants took less time to initiate their search in their successful search trials as compared to unsuccessful search trials may indicate memory retrieval i.e., infants’ attempt to recall resulting in failure in unsuccessful search trials. It is also possible that it may be interpreted as indicating infants’ uncertainty. There is some evidence that confidence judgments in adults converge with response latency (e.g., Koriat and Sorka, 2015; Weidemann and Kahana, 2016) and answer fluency serves as a metacognitive cue (e.g., Bjork et al., 2013 for a review). Although not statistically significant (note the small number of infants), the final set of analyses indicates that a comparatively larger number of infants succeeded in the short onset delay group as compared to the long onset delay group especially in the one-box and in the two-box trial. Infants’ delay in search initiation in the present research, therefore, may indicate their pre-decisional uncertainty, in contrast with the post-decisional uncertainty as investigated by Goupil and Kouider (2016). Notably, the three trial types used in the present study are likely to involve different kinds of relationship between searching and onset delay. Successful searching is entirely based on guessing in the three-box trial, memory retrieval in the one-box, and both guessing and memory in the two-box trial. Our data, however, do not clearly determine the kinds of relationship between searching success and onset delay involved (and a different cognitive process) in each task type. Additionally, in the two-box trial infants’ unsuccessful searching involves either an accurate search error (if infants chose one of the initial two boxes present at hiding) or an inaccurate one (if they chose the box not present at hiding but added later). Our sample size was not big enough to analyze these different search error patterns in their relationship to the onset delay and a future investigation into this issue will be able to answer different cognitive processes involved. A systematic investigation of a relationship between response latency and memory recall in infants, especially using multiple trials per uncertainty task type, therefore, will greatly contribute to the discussion of the nature of infants’ epistemic sensitivity to uncertainty.

In light of the debate on the ontogeny of metacognition, a working hypothesis is that naturally occurring behaviors expressing uncertainty – such as response reluctance – may be recruited as predictive signals, helping children to become able to use uncertainty monitoring in order to control their cognitive decisions. Whether such uncertainty cues are the basis of experience-based feelings of confidence (see Koriat, 1993) allowing people to reliably adjust their own behaviors should be more systematically investigated in future – preferably longitudinal – studies. It has been proposed that the basis of metacognitive abilities – albeit rudimentary – are likely to develop early in human development (Goupil et al., 2016). An interesting possibility is that the kinds of reluctance or appetence expressed in our study (and sensitivity to others’ as well as one’s own reluctance or appetence to respond) may first develop to support and facilitate learning and communication, well before more sophisticated forms of language-dependent metacognitive abilities develop, by serving as cues for their own uncertainty to further control their own behaviors (e.g., information seeking behavior as in Goupil et al., 2016 or Call and Carpenter, 2001) and also by signaling lack of confidence to others (i.e., communicative partners and teachers). Overall, to what extent and by what exact mechanisms reluctance is used in uncertainty monitoring in young children and might contribute to the emergence of metacognition deserves more investigations (see also Kepecs and Mainen, 2012).

Our study also provides some insight into infants’ searching literature. In fact, searching for a toy that has just gone out of sight is not an easy task. It requires an extended developmental trajectory of experiences and cognitive and motor maturity and a coordination of both (Corbetta et al., 2018). Infants fail to manually search for an out-of-sight object until around 8 months old (Piaget, 1954; see Vishton, 2018 for a review). In addition, infants reliably search for an object that has gone out of their sight due to darkness earlier than due to occlusion (e.g., Shinskey and Munakata, 2003). Infants’ searching also informs us about their ability to individuate objects (Van de Walle et al., 2000) or understand the concept of an object’s support event (Hespos and Baillargeon, 2008). For example, Van de Walle et al. (2000) demonstrated that 12-month-olds infants reached for an object inside an empty container more frequently and searched longer when they believed that an additional object was hidden after retrieving a first object than when they believed that only a single object was hidden. Together, infants’ searching behaviors inform us about infants’ object knowledge and the cognitive processes associated to it. The present findings speak to this literature by demonstrating that infants are able to combine their object knowledge (e.g., object permanence) to appropriately search for the hidden object under probabilistic information of a hiding place.

Finally, we need to acknowledge some limitations in our experiments. First, our procedure may be complicated, taxing infants’ memory capacity, especially that of 12-month-olds. For example, infants were required to continue to remember in which container the toy had been hidden while additional boxes were added behind the screen in the one box and two box tasks. Yet, it is noteworthy that in the three-box task which is less demanding in this respect (no need to keep track of the hiding place), infants’ reluctance increased compared to the other two tasks. Secondly, due to infants’ short attention span, we administered only one trial per probability task type (a total of three trials), which precludes us from observing infants’ consistent behaviors across trials. Nevertheless, we hope that our study provides first steps toward exploring infants’ searching time initiation as a way to investigate infants’ reaction to uncertainty and probabilistic reasoning – and potentially their metacognitive sensitivity.

## Data Availability Statement

All datasets generated for this study are included in the Supplementary Material. The raw data are available in Supplementary Data Sheet 1.

## Ethics Statement

The studies involving human participants were reviewed and approved by the Ludwig Maximilian University of Munich Ethic Committee. Written informed consent to participate in this study was provided by the participants’ legal guardian/next of kin.

## Author Contributions

SK and JP contributed conception and design of the study. SK performed the statistical analysis. SK, BS, and JP wrote the manuscript. All authors contributed to manuscript revision, read and approved the submitted version.

## Conflict of Interest

The authors declare that the research was conducted in the absence of any commercial or financial relationships that could be construed as a potential conflict of interest.

## Footnotes

1 Chance was set at 0.33, selecting one out of three boxes.
